# Migraine patients’ journey until a tertiary headache center: an observational study

**DOI:** 10.1186/s10194-019-1039-3

**Published:** 2019-08-15

**Authors:** Mario Fernando Prieto Peres, Diego Belandrino Swerts, Arão Belitardo de Oliveira, Raimundo Pereira Silva-Neto

**Affiliations:** 1 Hospital Israelita Albert Einstein, São Paulo, Brazil. Instituto de Psiquiatria, Hospital das Clínicas da Faculdade de Medicina da USP, Rua Joaquim Eugênio de Lima, 881 cj 708, Sao Paulo, SP Brazil; 2 Faculdade de Medicina do Hospital Israelita Albert Einstein, São Paulo, Brazil; 3 Instituto de Psiquiatria, Hospital das Clínicas da Faculdade de Medicina da USP, Sao Paulo, Brazil; 4 Universidade Federal do Piauí, Disciplina de Neurologia, Teresina, PI Brazil

**Keywords:** Migraine disorders, Burden, delayed diagnosis, Tertiary healthcare, Time-to-treatment

## Abstract

**Background:**

Migraine diagnosis is based on clinical aspects and is dependent on the experience of the attending physician. This study aimed to describe the patients journey profile until they start their experience in a tertiary headache center.

**Methods:**

In a cross-sectional study, medical charts from migraine patients were reviewed to describe which treatments, procedures and follow-up strategies are performed until the first appointment with a headache specialist. Patients from both sexes, ≥18 years old, which came to their first visit from March to July 2017 were included. Sociodemographic information, headache characteristics, diagnostic methods previously used, clinical history, family history and the treatments previously used were assessed in the first appointment with a specialist. Patient Health Questionnaire-9 and General Anxiety Disorder-7 were also applied. Descriptive analyses were performed to describe the sample profile and statistical tests were used to evaluate factors associated with the type of migraine (chronic or episodic).

**Results:**

The sample consisted of 465 patients. On average, the pain started 17.1 (SD = 11.4) years before the first appointment with a headache specialist. Most of patients were classified as having chronic migraine (51.7%), with an average frequency of 15.5 (SD = 9.9) days per month. Regarding patients’ journey until a specialist, most patients were submitted to laboratory tests (74.0%), cranial tomography (66.8%) and magnetic resonance imaging (66.8%) as diagnostic methods, and preventive drugs (70.2%) and acupuncture (61.0%) as treatments. After stratification by migraine type as episodic or chronic, patients with chronic migraine were submitted to more magnetic resonance imaging test, acupuncture, psychotherapy, used preventive drugs, and reported to have used topiramate without beneficial effects.

**Conclusions:**

Brazilian patients with migraine experiment a long journey until getting to a headache specialist and are submitted to a great number of unnecessary exams, especially those with chronic migraine.

## Background

Headache is one of the leading causes of need for medical care, according to the World Health Organization (WHO) [1]. Estimated worldwide prevalence is in average 12% and in countries from Central and South America, 16% [2]. In Brazil, the prevalence estimates may vary with the type of headache from 15.2% for migraine to 70.6%, for any headache type [3].

Migraine is characterized by recurrent attacks of moderate to severe, usually with pulsatile and unilateral headache, and is one of the most disabling diseases [4]. It is a multifactorial disease, with genetic, endogenous and environmental factors. Frequency may vary from episodic to a daily basis [5]. Chronic migraine is defined by the occurrence of episodes of headache in at least 15 days per month, for at least three months, and the pain has characteristics of migraine (unilateral aspect, pulsatile, moderate or severe pain, presence or absence of aura, and others) in at least eight days per month [6, 7]. Chronic migraine is significantly associated with highest degree of disabilities when compared with episodic migraine [8].

Although migraine sufferers experience often disable headaches, a significant proportion never consulted a physician, general practitioner, neurologist or a tertiary headache specialist [9, 10]. Disease burden elevates with poor health care access.

More attention has been given lately to the patient journey as an attempt to improve health care quality [11]. But limited information is available on the journeys patients take before reaching one tertiary headache center [12]. In the UK, 200 patients were studied on their first attendance at a headache clinic. Most patients had not been given a formal diagnosis in primary care, and only a few patients had been offered triptans [13]. In a similar pattern, an Italian survey has shown that more than 70% of patients receive the diagnosis of migraine when attending to a headache center and only 26.8% had a previous diagnosis of the condition [14].

We aimed to describe in this study the patients journey profile until they start their experience in a tertiary headache center, so we could deeply understand patients need, improving headache specialized care.

## Methods

### Study design

This was a single-center, observational, cross-sectional study conducted in order to describe which treatment, procedures and any follow-up strategies migraine patients in Brazil have performed until the first appointment with a headache specialist in a tertiary center. Information were extracted from medical charts of patients who answered to an interview during the first medical visit at the study site. The study was conducted in accordance with local laws. Since it was a cross-sectional study conducted by medical charts review, without subjects’ identification, there was no need to sign an informed consent.

### Eligibility criteria

Medical charts from adult patients from both sexes, 18 years old or higher, who came to their first visit in a tertiary headache center, in Sao Paulo (Sao Paulo Headache Center), were included in the analysis, from March to July 2017. Exclusion criteria were: men or women below the age of 18, patients with associated dementia, or significant neurological deficit.

### Study procedures

At Sao Paulo Headache Center patients are usually submitted to an interview during the first routine visit. The questionnaire contains questions on patients’ sociodemographic information, headache characteristics, diagnostic methods previously used, clinical history, family history and treatments previously used. Two standardized instruments are used: Patient Health Questionnaire-9 (PHQ-9) and General Anxiety Disorder-7 (GAD-7).

PHQ-9 is composed by nine questions to assess the severity of depression through the presence of the following symptoms in the past two weeks: depressed mood, anhedonia, having trouble sleeping, feeling tired, change in appetite or weight, feelings of guilt and worthlessness, having trouble concentrating, feeling slowed down or restless, and having suicidal thoughts. The answers are given in a 4-point Likert scale in which patients chose between “not at all”, “several days”, “more than half the days” and “nearly every day”. Final scores are calculated through the sum of each answer and stratified in five groups of depression severity (minimal or none: 0–4; mild: 5–9; moderate: 10–14; moderately severe: 15–19; severe: 20–27) [15].

GAD-7 is an instrument used for assessment, diagnosis and monitoring of anxiety. It is composed by seven items, disposed in a 4-point Likert scale (0: not at all; 3: nearly every day). Final scores are calculated through the sum of each answer and stratified into four severity groups (minimal/no anxiety: 0–4; mild: 5–9; moderate: 10–14; severe: 15–21) [16].

### Statistical analysis

A descriptive analysis was performed to describe the sample profile. Measures of central tendency and dispersion were used for numerical variables and measures of frequency for categorical variables.

Chronic and episodic migraine were defined based on the International Classification of Headache Disorders [7]. In order to evaluate factors associated with the type of migraine (chronic or episodic) statistical tests were performed. Categorical variables’ association was assessed using the Chi Square test. Numeric variables relationship was assessed using T test (variable with normal distribution) or Mann-Whitney (variable without normal distribution) test. The data normality was identified by Shapiro-Wilk test. Statistically significant results were tabulated. A significance level of 5% was adopted and the analyses were performed in statistical software R Project Version 3.5.1.

## Results

### Patients’ profile

The sample consisted of 465 patients and their profile is shown in Table 1. Of the total sample, 72.7% were women, 58.9% were married and 37.8% were employed. Mean age was 37.3 years old (SD = 13.0) and Body Mass Index 24.1 (SD = 3.9).

**Table 1:** Sociodemographic and clinical characteristics of patients

Sociodemographic and clinical data	N	%
Gender (*n* = 462)		
Men	126	27.3
Women	336	72.7
Marital status (*n* = 241)		
Married	142	58.9
Single	79	32.8
Divorced	16	6.6
Widower	3	1.2
Separated	1	0.4
Activity/main occupation (*n* = 230)		
Employed	176	76.5
Student	37	16.1
Housewife	14	6.1
Retired	2	0.9
Unemployed	1	0.4
Age (Mean/SD - *n* = 463)	37.3	13.0
BMI (Mean/SD - *n* = 334)	24.1	3.9
Patient Health Questionnaire - 9 (PHQ-9) (*n* = 445)		
Minimal or none	152	34.2
Mild	152	34.2
Moderate	75	16.9
Moderately severe	35	7.9
Severe	31	7.0
General Anxiety Disorder - 7 (GAD-7) (n = 445)		
None	139	31.2
Mild	144	32.4
Moderate	93	20.9
Severe	69	15.5
Health problems (*n* = 355)		
Rhinitis	180	50.7
Sinusitis	175	49.3
Gastritis	173	48.7
Kidney Stone	66	18.6
Polycystic ovary	58	16.3
Systemic arterial hypertension	39	11.0
Endometriosis	22	6.2
Fibromyalgia	21	5.9
Family member who also had a history of headache (*n* = 207)		
Mother	118	57.0
Siblings	71	34.3
Grandparents	53	25.6
Father	49	23.7
Children	25	12.1

*SD* Standard deviation

Through PHQ-9 analysis, 68.4% of patients were classified with minimal/none or mild depression. GAD-7 results showed 63.6% of patients classified as having no symptoms or mild symptoms.

The three main health problems reported by the patients were rhinitis (38.7%), sinusitis (37.6%) and gastritis (37.2%). The most frequently reported family member who also had a history of headache was mother (25.4%).

Headache characteristics are presented in Table 2. On average, patients reported the beginning of pain 17.1 (SD = 11.4) years before the interview, attack duration of 53.7 (SD = 62.0) hours and a pain intensity of 6.8 in a 0–10 scale. Most cases were classified as chronic migraine (51.7%). Mean headache frequency was 15.5 (SD = 9.9) days per month. Furthermore, 29.9% of the patients reported migraine with aura.

**Table 2:** Headache characteristics reported by patients

Headache characteristics	N	%
History of Headache in Years (Mean/SD - *n* = 459)	17.1	11.4
Pain intensity (0–10) (Mean/SD - *n* = 464)	6.8	2.1
Mean attack duration (hours) (Mean/SD - *n* = 216)	53.7	62.0
Mean headache frequency per month (days) (Mean/SD - n = 464)	15.5	9.9
Episodic migraine (up to 14 days)	224	48.3
Chronic migraine (15 or more days)	240	51.7
Migraine with aura (*n* = 448)		
Yes	134	29.9
No	314	70.1
Time of pain onset (*n* = 464)		
Morning	307	66.2
Afternoon	310	66.8
Night	280	60.3
Dawn	140	30.2
Head side affected by pain (*n* = 464)		
Bilateral	189	40.7
Unilateral/alternating	179	38.6
Only left side	50	10.8
Only right side	46	9.9
Pain location (*n* = 464)		
Ocular	240	51.7
Temporal	226	48.7
Frontal	206	44.4
Occipital	201	43.3
Parietal	187	40.3
Vertex	120	25.9
Neck	110	23.7
Type of pain (*n* = 464)		
Throbbing	331	71.3
Dull/Aching	226	48.7
Others	29	6.3

*SD* Standard deviation

In 59.3% of patients, headache location was unilateral. Regarding pain location, ocular region was the most reported (51.7%), followed by temporal (48.7%) and frontal (44.4%). Throbbing type headache was described in 71.3% of patients.

### Patients’ journey until specialist

Laboratory tests (74.0%), computerized tomography (66.8%) and magnetic resonance imaging (66.8%) were previously used as diagnostic methods by more than half of patients (Table 3).

**Table 3:** Previous methods of diagnosis reported by patients

Previous methods of diagnosis	N	%
Tests (*n* = 392)		
Laboratory tests	290	74.0
Cranial tomography	262	66.8
Magnetic resonance imaging	262	66.8
Electroencephalogram	163	41.6
Others	21	5.4

Regarding treatments previously prescribed to patients, the most frequently used were preventive drugs and acupuncture, in 70.2% and 61.0% of patients, respectively. Other reported treatments were: psychotherapy (*n* = 178; 48.2%); physiotherapy (*n* = 129; 36.4%); anesthetic blockages (*n* = 91; 26.1%); meditation (*n* = 75; 21.1%); botulinum toxin (*n* = 67; 19.1%); and biofeedback (*n* = 4; 1.2%) (Table 4).

**Table 4:** Patients’ experience with non-pharmacological treatments

Treatments (*n* = 423)	Never used		Currently used		I did it without succeed		I did it with good results	
Acupuncture (*n* = 382)	149	39.0	40	10.5	129	33.8	64	16.8
Psychotherapy (*n* = 369)	191	51.8	67	18.2	52	14.1	59	16.0
Physiotherapy (*n* = 354)	225	63.6	22	6.2	48	13.6	59	16.7
Botulinum toxin (*n* = 350)	283	80.9	9	2.6	30	8.6	28	8.0
Preventive drugs (*n* = 386)	115	29.8	90	23.3	124	32.1	57	14.8
Anesthetic blockages (*n* = 349)	258	73.9	24	6.9	41	11.7	26	7.4
Biofeedback (*n* = 333)	329	98.8	2	0.6	–	–	2	0.6
Meditation (*n* = 355)	280	78.9	23	6.5	29	8.2	23	6.5

Patients’ experiences using preventive drugs and triptans/ergotamines are shown in Tables 5 and 6, respectively.

**Table 5:** Patients’ experience with preventive drugs

Preventive medicines (*n* = 465)	I have not used it for a long time		I have already used it without beneficial effect		I have already used it with beneficial effect but with side effects		Never used	
	N	%	N	%	N	%	N	%
Topiramate	49	28.8	75	44.1	75	44.1	295	63.4
Divalproate	28	31.5	48	53.9	18	20.2	376	80.9
Propranolol, Atenolol, Nadolol	36	31.3	54	47.0	26	22.6	350	75.3
Melatonin	38	49.4	30	39.0	10	13.0	388	83.4
Amitriptyline	32	33.3	46	47.9	22	22.9	369	79.4
Nortriptyline	29	30.9	52	55.3	19	20.2	371	79.8
Flunarizine	28	45.2	28	45.2	8	12.9	403	86.7
Fluoxetine	31	35.2	35	39.8	25	28.4	377	81.1
Sertraline	24	42.1	22	38.6	11	19.3	408	87.7
Venlafaxine	23	38.3	23	38.3	15	25.0	405	87.1
Desvenlafaxine	25	64.1	9	23.1	5	12.8	426	91.6

**Table 6:** Patients’ experience with triptans/ergotamines

	Does not work		Has regular effect/Does not always work		Has good effect		Never used	
	N	%	N	%	N	%	N	%
Naratriptan hydrochloride	51	28,5	81	45,3	47	26,3	286	61,5
Zolmitriptan	28	44,4	20	31,7	15	23,8	402	86,5
Rizatriptan benzoate	27	35,5	24	31,6	25	32,9	389	83,7
Sumatriptan succinate	55	32,4	51	30,0	64	37,6	295	63,4
Dihydroergotamine mesylate + dipyrone monohydrate + caffeine	65	48,9	47	35,3	21	15,8	332	71,4
Dihydroergotamine mesylate + paracetamol + caffeine + metoclopramide hydrochloride	74	62,2	35	29,4	10	8,4	346	74,4

Regarding preventive drugs, excluding patients who have not used the medication for a long time, all drugs were classified as “without beneficial effect” and “with beneficial effect, but with side effects”. Citalopram was classified as without beneficial effect by 14 of 17 patients (82.4%). In addition, flunarizine, melatonin and nortriptyline were reported with negative results, classified as without beneficial effects by 77.8%, 75.0% and 73.2% of the cases, respectively (Table 5). Of the 465 patients, 115 (24.7%) were using preventive drug at moment of information collection.

About triptans/ergotamines used, sumatriptan succinate was the most effective drug (37.6% of the patients reporting a good effect), followed by rizatriptan (32.9%) and naratriptan hydrochloride (26.3%). The combination of dihydroergotamine mesylate, paracetamol, caffeine, and metoclopramide hydrochloride presented the worst evaluation, with 62.2% of reports as ‘does not work’, 29.4% of ‘regular effect or might not work’ and only 8.4% of ‘good effect’.

The study sample was stratified by the type of migraine, episodic or chronic, to assess association with several characteristics of patients’ journey. Table 7 shows those who had statistically significant results. Patients with chronic migraine were submitted to more magnetic resonance imaging test, acupuncture, psychotherapy, preventive drugs, and reported to have used topiramate without beneficial effects. The use of anesthetic blockages was more frequent among patients with episodic migraine.

**Table 7:** Characteristics of patients’ journey significantly associated with the type of migraine

		Migraine				*p*-value
		Episodic		Chronic		
		N	%	N	%	
Test - Magnetic resonance imaging	No	116	51.8	86	35.8	0.001
	Yes	108	48.2	154	64.2	
Treatment – Acupuncture	No	88	47.8	61	30.8	0.001
	Yes	96	52.2	137	69.2	
Treatment – Psychotherapy	No	104	57.8	87	46.0	0.031
	Yes	76	42.2	102	54.0	
Treatment - Preventive medicines	No	73	39.2	42	21.0	< 0.001
	Yes	113	60.8	158	79.0	
Treatment - Anesthetic blockages	No	136	81.9	122	66.7	0.002
	Yes	30	18.1	61	33.3	
Preventive drug - Topiramate	I have not used it for a long time	25	41.0	24	23.1	0.001
	I have already used it without beneficial effect	14	23.0	56	53.8	
	I have already used it with beneficial effect but with side effects	22	36.1	24	23.1	

## Discussion

This study was conducted with the primary aim to describe the journey of patients with migraine in a tertiary headache center in Sao Paulo, Brazil until their first appointment. In order to answer this objective, medical charts from patients who attended to a tertiary headache center were reviewed, collecting information about patients’ history registered in the first attendance. As main results, diagnostic methods, treatments and preventive strategies previously used by patients were reported.

More than half of patients reported to have previously performed laboratory tests, cranial tomography and magnetic resonance imaging. However, guidelines do not recommend the use of any medical exams for migraine diagnosis [17–19]. The use of unnecessary strategies to investigate signs and symptoms may have different impacts, especially for patients, such as the exposure radiation present in tomography and magnetic resonance imaging. Viana et al. (2016) have shown that most patients also perform a great number of unnecessary exams, of which 40% includes radiation exposure [20].

Regarding treatments previously performed, most patients used preventive drugs and acupuncture. The use of preventive drugs is in accordance with several guidelines which state that the cure is not the aim of migraine management. We have not listed the option of effective treatment without side effects. The medication list refers to previous experiences, we believe patients experiencing effective treatments without side effects are less likely to reach a tertiary headache center, or they may be fitting into the “I have not used for a long time” category. Especially in cases of chronic disease, treatment has the objective to reduce frequency and intensity of crises [17–19]. The use of alternative therapies is not recommended due the lack of evidence on its efficacy in migraine treatment. Only acupuncture has shown satisfactory results when compared to topiramate in a clinical trial and is recommended by the Latin American consensus for the treatment of chronic migraine [21, 22]. Thus, although prescribed by nonspecialized assistance, the treatment seems to be in accordance with recommendations.

Vincent and colleagues (1999) have previously reported the primary headache care delivered by nonspecialists in Brazil. In order to assess this objective, the study interviewed 414 patients with questions such as the duration of headache, the need for medical assistance, types of diagnoses provided by nonspecialists, the sort of investigations and treatments prescribed. Patients reported that had seen on average 3 health assistants before the appointment with a specialist and a headache beginning on average 11 years before. As observed in the present study, patients performed a great number of investigative procedures, such as electroencephalography, computerized tomography, and sinus and skull x-rays. Regarding prescribed treatment, a prophylactic strategy was adopted by 48.9% of migraine patients, involving benzodiazepines, tricyclic antidepressants, beta-blockers, flunarizine, anticonvulsants, pizotifen and nonpharmacologic strategies such as use of new glasses, diet and homeopathic treatment [23]. These data are consistent with those presented in this study, showing that migraine patients are benefited from specialized care and that misdiagnosis is still observed twenty years later. Furthermore, data suggests the use of a large amount of unnecessary health resources by migraine patients, generating burden to both, patients and society. The need for conduction of a study estimating economic burden of migraine in Brazil, to assess the real impact of these practices, is highlighted.

Patients’ experience about drugs used in the treatment and as a preventive approach was assessed, describing how they classify medicines regarding their efficacy and the occurrence of side effects. Preventive drugs were classified by patients as without beneficial effects or when the beneficial effects are observed, side effects are also reported. The preference of Brazilian patients for migraine preventive therapy was previously assessed and the effectiveness was the most important aspect of the treatment by 92.7% of the sample. The occurrence of adverse events was the third of seven aspects in the classification of most important, by 32.4% of the patients [24]. These data reinforce the need for investment in technologies that combine both effectiveness and safety, which are of great concern of migraine patients.

The sample was further stratified by the type of migraine, as episodic or chronic. The prescription of magnetic resonance imaging test, acupuncture, psychotherapy, use of preventive drugs and use of topiramate without beneficial effects was most likely to be observed among those with chronic disease. The use of anesthetic blockages was more frequent among patients with episodic disease. To date, this is the first study to assess differences in the treatment performed before the attendance with specialist comparing patients with different types of migraine. When resource utilization is compared, chronic migraine patients seems to report more visits to general practitioners, neurologists, nurse and physician assistant, diagnostic testing and other exams and the use of drugs [25]. So, the journey of patients, stratified by the type of migraine, still needs to be further addressed by other studies to confirm the associations found.

Other outcomes were reported by the study, including the presence of anxiety and depression, through the standardized instruments GAD-7 and PHQ-9. A greater frequency of anxiety was observed when compared with depression symptoms, once 68.8% of patients presented some level of anxiety and 66%, some level of depression. This result corroborates the association between anxiety and migraine, more robust than depression, as reported in a Brazilian study recently published [26]. Other results such as sociodemographic characteristics are consistent with those published in the country [26, 27].

Although international guidelines states that most primary and medication-overuse headaches may be adequately managed on primary healthcare, the same reports also highlight the cases where referral to a specialist is necessary [28]. This need for referral is reinforced by data shown in the present study.

Important to add the referral pattern, as previous migraine diagnosis. Patients come to our headache center by self-referral, and the vast majority had already a migraine diagnosis.

This study has several limitations, specially related to the source of information. When using information from medical charts, data is dependent on the quality of registration which may compromise the available reports. Another limitation is the retrospective nature of the interview, in which patients were asked to remember all the procedures previously performed. However, this study adds important knowledge about treatment of migraine in Brazil.

## Conclusion

Brazilian patients with migraine experiment a long journey until getting to a headache specialist, which may last on average 17 years since the first headache episode. These patients are submitted to a great number of unnecessary exams, although diagnosis of migraine may be performed only based on clinical examination and the evaluation of patients’ history. However, the treatment performed by non-specialists seems to be consistent with that proposed by guidelines. When migraine patients are stratified in chronic and episodic cases, the use of unnecessary technologies is still greater in those with chronic disease.

This study reinforces the need of a specialized assistance for migraine patients, so patients have more assertive treatment, improving impact of the disease and quality of life.

## Data Availability

Data is available upon reasonable request and with permission of Sao Paulo Headache Center.
